# Deletion of Calhm2 alleviates MPTP-induced Parkinson's disease pathology by inhibiting EFHD2-STAT3 signaling in microglia

**DOI:** 10.7150/thno.83082

**Published:** 2023-03-13

**Authors:** Xuena Bo, Fei Xie, Jingdan Zhang, Runze Gu, Xiaoheng Li, Shuoshuo Li, Zengqiang Yuan, Jinbo Cheng

**Affiliations:** 1 Center on Translational Neuroscience, College of Life & Environmental Science, Minzu University of China, Beijing, 100081, China; 2 The Brain Science Center, Beijing Institute of Basic Medical Sciences, Beijing, 100850, China; 3 School of Basic Medical Sciences, Anhui Medical University, Hefei, 230032, China

**Keywords:** Microglia, Calhm2, Parkinson's disease, Neuroinflammation, Pathology

## Abstract

**Background:** Neuroinflammation is involved in the development of Parkinson's disease (PD). Calhm2 plays an important role in the development of microglial inflammation, but whether Calhm2 is involved in PD and its regulatory mechanisms are unclear.

**Methods:** To study the role of Calhm2 in the development of PD, we utilized conventional Calhm2 knockout mice, microglial Calhm2 knockout mice and neuronal Calhm2 knockout mice, and established the MPTP-induced PD mice model. Moreover, a series of methods including behavioral test, immunohistochemistry, immunofluorescence, real-time polymerase chain reaction, western blot, mass spectrometry analysis and co-immunoprecipitation were utilized to study the regulatory mechanisms.

**Results:** We found that both conventional Calhm2 knockout and microglial Calhm2 knockout significantly reduced dopaminergic neuronal loss, and decreased microglial numbers, thereby improving locomotor performance in PD model mice. Mechanistically, we found that Calhm2 interacted with EFhd2 and regulated downstream STAT3 signaling in microglia. Knockdown of Calhm2 or EFhd2 both inhibited downstream STAT3 signaling and inflammatory cytokine levels in microglia.

**Conclusion:** We demonstrate the important role of Calhm2 in microglial activation and the pathology of PD, thus providing a potential therapeutic target for microglia-mediated neuroinflammation-related diseases.

## Introduction

Parkinson's disease (PD) is the second most common age-related neurodegenerative disease, and has been described as a movement disorder 1. Typical motor features of the disease include bradykinesia, limb stiffness, and tremor. The main pathological features are the progressive loss of dopaminergic (DA) neurons in the substantia nigra (SNc) and the formation of Lewy bodies due to the aggregation of alpha-synuclein 2, 3. A community-based study in China showed a PD prevalence of 1.37% in people aged > 60 years 4. The total number of people with PD in China is estimate to be as high as 3.62 million, posing a major challenge in a rapidly aging population 4. Risk factors in the development of Parkinson's disease include age, genetics, environment, immune status, and sex 5-7. The discovery of family mutations that cause PD, such as alpha-synuclein, parkin, PINK1, DJ-1, LRRK2 and ATP13A2 genes, has led to a better understanding of PD 8, 9. However, the majority (95%) of PD cases are disseminated and are thought to result from a complex interaction between genetic susceptibility and environmental factors 10. Recent studies suggest that inflammation acts as a bridge between the two and is involved in the development of PD 10, 11.

In 1988, McGeer et al. detected the presence of large numbers of HLA-DR-positive reactive microglia in the SNc of PD patients, which was one of the first pieces of evidence linking neuroinflammation to the pathogenesis of PD 12. Microglia—resident macrophages in the brain—account for 5-10% of all human brain cells and act as immune monitors of cellular surroundings 13. Normally, alpha-synuclein aggregates are released from neurons via extracellular secretion, and extracellular alpha-synuclein can diffuse into microglia via intercellular transfer or non-cell-autonomous means 14, 15. Microglia are unusually sensitive to cell-damage signals generated by dysfunctional neurons. These signals cause them to change from a resting state under healthy conditions to an activated state. Activated microglia exhibit two types of polarization: in the pro-inflammatory stage, microglia secrete large amounts of pro-inflammatory and chemokines, and in the anti-inflammatory stage, they exhibit increased phagocytosis and secrete anti-inflammatory cytokines that play an important role in neuroprotection and tissue repair 16. Alpha-synuclein disrupts the dynamic balance of microglial polarization by inhibiting microglial autophagy, accompanied by accelerated expression of pro-inflammatory cytokines 17. This causes irreversible damage to dopamine neurons, which in turn activates microglia, creating a vicious cycle within the SNc of the brain.

Calcium ions are important secondary messengers for cellular function and are stored in the endoplasmic reticulum and mitochondria. Dysregulation of calcium homeostasis is a key feature of PD pathogenesis 18. In models of PD induced by mutations in the genetic factor GBA1, mutant neurons exhibit dysregulation of calcium homeostasis and increased vulnerability to stress responses, including elevated cytoplasmic calcium levels 19. PINK1 deficiency causes mitochondrial accumulation of calcium, resulting in mitochondrial calcium overload 20. L-Ornithine L-Aspartate was found to slow the progression of PD by regulating intra-mitochondrial calcium homeostasis and restoring mitochondrial function in a PD model 21. Additionally, Rifampicin has been found to reduce neuroinflammation by reducing mitochondrial dysfunction due to impaired calcium homeostasis in PD 22. The Calcium Homeostasis Modulator (CALHM) family consists of Calhm1 to Calhm6. Calhm1 is the most intensively studied member of this group, and the Calhm1 P86L single nucleotide polymorphism has also been suggested as a risk factor for Alzheimer's disease (AD) 23. Our previous study found that Calhm2 expression was increased in AD model mice, and that knockout of Calhm2 improved Aβ plaque deposition, reduced neuroinflammation, and improved cognitive impairment in AD model mice. Further studies revealed that microglia-specific knockout of Calhm2 also improved pathological manifestations in AD mice, and both *in vivo* and *in vitro* experiments demonstrated that inhibition of Calhm2 significantly inhibited microglial activation 24. Calhm2 plays an important role in the development of microglial inflammation, but whether Calhm2 is involved in PD and its regulatory mechanisms are unclear.

In the present study, we found that Calhm2 plays an important role in the 1-Methyl-4-phenyl-1,2,3,6-tetrahydropyridine (MPTP)-induced mouse model of PD. Either conventional knockout of Calhm2 or microglial conditional knockout of Calhm2 rescued the MPTP-induced loss of tyrosine hydroxylase (TH)-positive neurons and inhibited neuroinflammation, thereby improving motor deficits in PD model mice.

## Results

### Conventional knockout of Calhm2 improves locomotor performance, and reduces DA neuronal loss and microglia numbers

**Figure 1:**
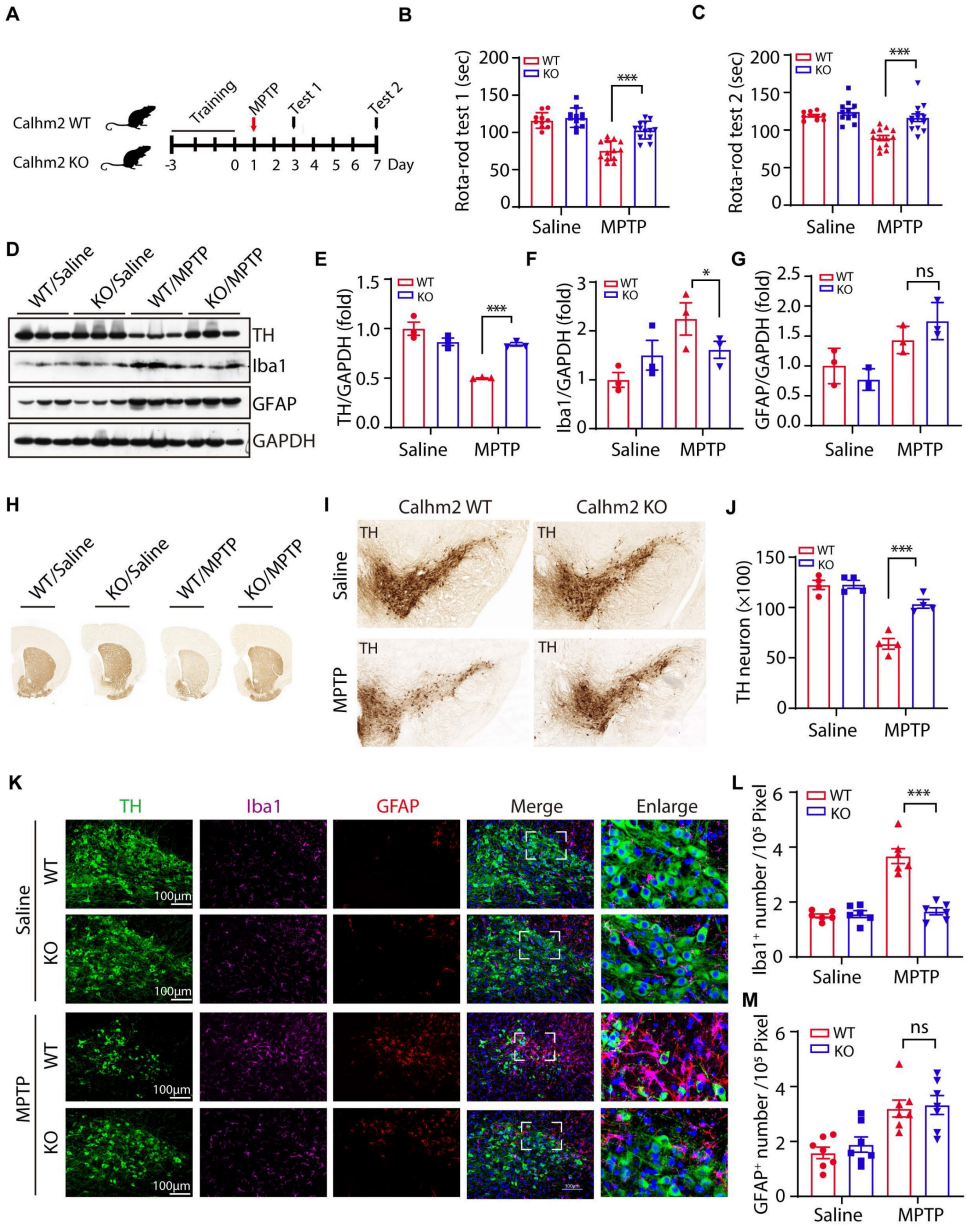
** Conventional knockout of Calhm2 improves locomotor performance, reduces DA neuronal loss and microglia numbers.** (A) Calhm2 WT and Calhm2 KO mice were treated with saline or MPTP (four intraperitoneal injections of 20 mg/kg, 2 h intervals) after three days of rotarod training. Behavioral tests were performed on the third and seventh day after MPTP injection and striatal tissue was collected on the seventh day. (B and C) Latency of mice on the rotating rod. Data are expressed as mean ± S.E.M. (ANOVA, *P < 0.05, **P < 0.01, ***P < 0.001, n ≥ 10) (D-G) Immunoblotting and statistical analysis of Striatal tissue lysates using antibodies against Iba1, GFAP, and TH after MPTP treatment (n = 3). (H) Images of immunohistochemically stained sections of mouse striatum treated with anti-TH antibody. (I) Images of immunohistochemically stained sections of TH in the SNc of mice. (J) Statistical analysis of the number of TH-positive neurons in the SNc of mice. Data are expressed as mean ± S.E.M. *P < 0.05, **P < 0.01, ***P < 0.001, n = 4. (K) Co-localization of TH (immunostaining) with Iba1 (immunostaining) and GFAP (immunostaining) positive cells in Calhm2 WT and Calhm2 KO mice. (L, M) Statistical analysis of the number of microglia as well as astrocytes. *P < 0.05, **P < 0.01, ***P < 0.001, (n = 6 slices for 3 mice per group).

We previously reported that Calhm2 regulated microglial activation-mediated neuroinflammation and played an important role in the pathology of Alzheimer's disease 24. However, the mechanism by which Calhm2 regulates microglial activation and whether Calhm2 is involved in other neuroinflammation-related diseases was unclear. In this study, we investigated the role of Calhm2 in MPTP-induced PD and its underlying mechanisms. To that end, Calhm2 knockout mice (Calhm2 KO) and Calhm2 wide-type mice (Calhm2 WT) were used to generate MPTP-induced PD mice model (**Figure S1A**). The mice were subjected to three days of rotarod training, followed by MPTP injection (four intraperitoneal injections at 2 h intervals of 20 mg/kg each in one day), and behavioral testing after 3 and 7 days of MPTP treatment (**Figure 1A**). The results of the first rotarod test showed that after 3 days of MPTP treatment, mice had a significantly lower latency on the cylinder than mice injected with saline, indicating that MPTP induced PD symptoms, resulting in impaired locomotion in mice. Interestingly, knockout of Calhm2 was found to rescue this phenotype. Consistently, the results of the second rotarod test showed that after 7 days of MPTP treatment, Calhm2 KO mice showed significant enhancement in locomotor performance compared to Calhm2 WT mice (**Figure 1B-C**), suggesting that knockout of Calhm2 was beneficial for MPTP treatment-induced PD symptoms in mice.

Next, we examined whether knockout of Calhm2 altered TH levels in the brain and found that MPTP treatment resulted in a significant reduction in TH protein levels in the striatum compared to the control group, and that Calhm2 knockout significantly restored TH expression levels, suggesting that Calhm2 knockout could restore the loss of TH-positive neurons caused by MPTP treatment. We also examined the expression levels of glial fibrillary acidic protein (GFAP, an astrocyte marker) and ionized calcium-binding adaptor molecule 1 (Iba1, a microglial marker) in the brain. We found that MPTP treatment led to an increase in the expression levels of both GFAP and Iba1, and Calhm2 knockout inhibited Iba1 levels, but not GFAP levels (**Figure 1D-G**). The motor deficits in PD mice were associated with the loss of DA neurons in the SNc; therefore, we further investigated whether Calhm2 knockout could reduce MPTP-induced loss of DA neurons. The number of TH-positive neurons in the SNc was counted using immunohistochemical staining of TH. The results showed that MPTP treatment induced an approximately 50% loss of TH-positive neurons and that Calhm2 knockout significantly restored the loss of TH-positive neurons (**Figure 1H-J**).

Further, we performed immunofluorescence staining of glial cells. We found that under normal conditions, knockout of Calhm2 had no effect on either microglial, or astrocyte numbers. However, MPTP treatment increased both microglial and astrocyte numbers in the mouse brain. Interestingly, knockout of Calhm2 largely reduced the increase in microglial numbers but had no effect on astrocyte numbers in MPTP-treatment conditions (**Figure 1K-M**). Taken together, these results suggest that Calhm2 knockout improves locomotor performance and reduces DA neuronal loss and microglial numbers in PD model mice.

### Microglial specific Calhm2 knockout improves locomotor performance and reduces DA neuron loss in PD mice

**Figure 2:**
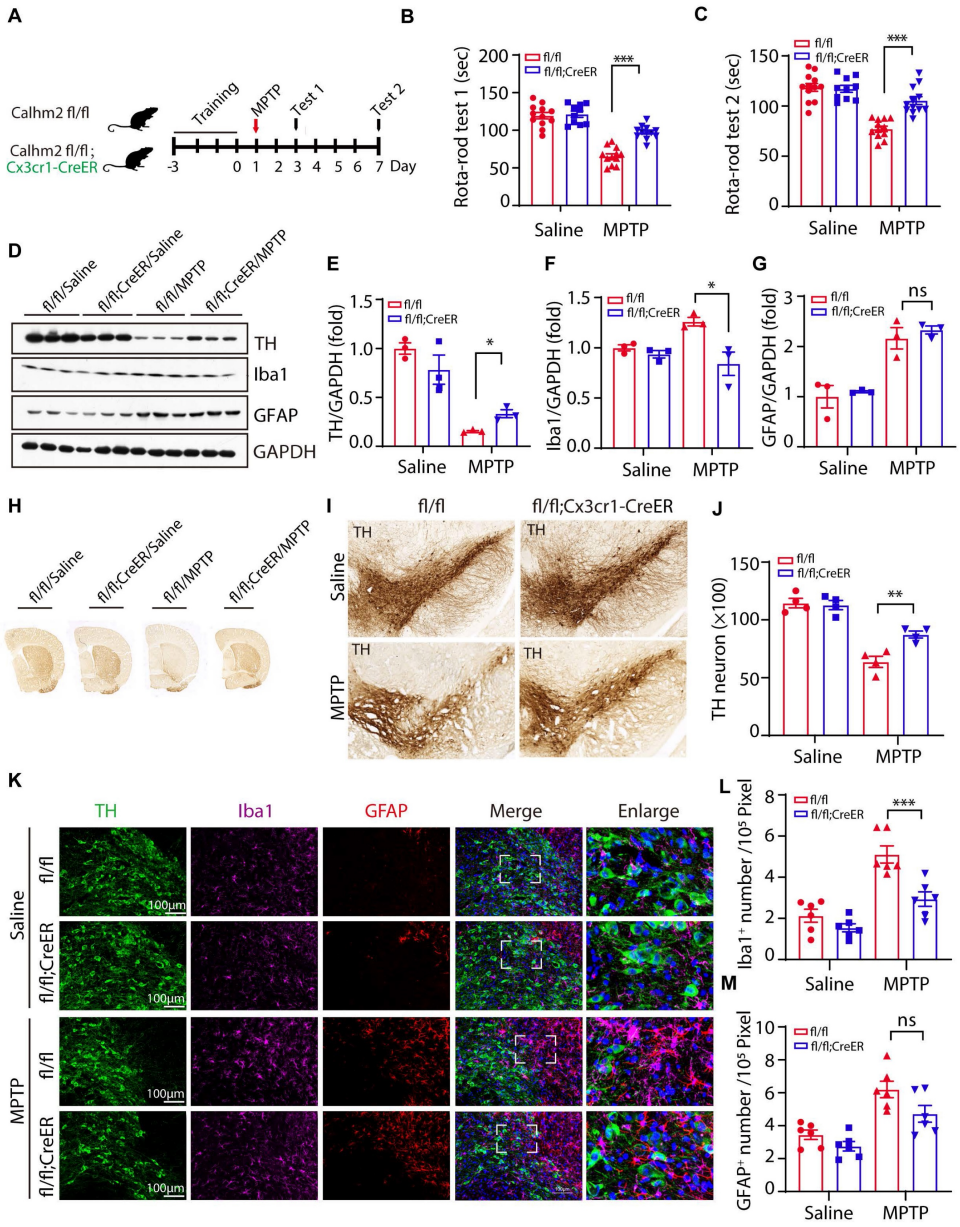
** Microglial specific Calhm2 knockout improves locomotor performance and reduces DA neuron loss in PD mice.** (A) Experimental flow diagram: 45-day-old Calhm2 flox/flox and Calhm2 flox/flox; Cx3cr1-CreER mice were administered a total dose of 20 mg of intragastric tamoxifen on three consecutive days. Mice were treated with saline or MPTP (four intraperitoneal injections of 20 mg/kg at 2 h intervals) after three days of rotarod training. Behavioral tests were performed on the third and seventh day after MPTP injection and striatal tissue was collected on the seventh day. (B, C) Latency of mice on the rotating rod. Data are expressed as mean ± S.E.M. (ANOVA, *P < 0.05, **P < 0.01, ***P < 0.001, n ≥ 10). (D-G) Immunoblotting and statistical analysis of Striatal tissue lysates using antibodies against Iba1, GFAP, and TH after MPTP treatment (n = 3). (H) Images of immunohistochemically stained sections of mouse striatum treated with anti-TH antibody. (I) Images of immunohistochemically stained sections of TH in the SNc of mice. (J) Statistical analysis of the number of TH-positive neurons in the SNc of mice. Data are expressed as mean ± S.E.M. *P < 0.05, **P < 0.01, ***P < 0.001, n = 4. (K) Co-localization of TH (immunostaining) with Iba1 (immunostaining) and GFAP (immunostaining) positive cells in Calhm2 flox/flox versus Calhm2 flox/flox;Cx3cr1-CreER mice. (L, M) Statistical analysis of the number of microglia as well as astrocytes. *P < 0.05, **P < 0.01, ***P < 0.001, (n = 6 slices for 3 mice per group).

The above results suggest that Calhm2 is involved in MPTP-induced loss of DA neurons and neuroinflammation. However, the cell type in which Calhm2 operates in the development of PD remains unclear. To investigate this, we crossed a Calhm2 flox/flox mouse strain with Cx3cr1-CreER transgenic mice to obtain a microglial conditional knockout of Calhm2 mice. Mice were gavaged with tamoxifen at 45 days after birth. Microglia were isolated from the brain one month later to confirm Calhm2 knockout efficiency (**Figure S2A**). After the three-day behavioral training of the rotarod, mice were treated with saline or MPTP separately (four intraperitoneal injections at 2 h intervals of 20 mg/kg each in one day). Behavioral tests of the rotarod were performed 3 and 7 days after MPTP injection, respectively. Additionally, pathological analyses were performed 7 days after MPTP injection (**Figure 2A**). The behavioral results showed that microglial conditional knockout of Calhm2 significantly protected against MPTP-induced motor deficits (**Figure 2B, C**). The loss of nigrostriatal DA neurons was always accompanied by the onset of motor deficits. We then performed immunoblotting experiments and immunohistochemical staining, and found that microglial Calhm2-specific knockout prevented the loss of TH protein levels and TH staining signals in the striatum after MPTP treatment (**Figure 2D-H**). Similarly, we stained TH-positive neurons in the SNc using immunohistochemical staining and found that microglia-specific knockout of Calhm2 significantly protected against MPTP-induced loss of TH-positive neurons (**Figure 2I, J**). At the same time, we examined the expression levels of GFAP and IbaI in the brain and found that MPTP injection resulted in elevated expression levels of GFAP (the astrocyte marker), and Iba1 (the microglia marker), and Calhm2 knockout showed a tendency to reduce the elevated Iba1 levels but not the elevated GFAP levels (**Figure 2D, F, G**). Furthermore, we performed immunofluorescence staining of GFAP and Iba1 in the SNc and found that microglial knockout of Calhm2 significantly reduced the increase in microglial numbers, but had no effect on astrocyte numbers under MPTP-treatment conditions (**Figure 2K-M**). Taken together, these results show that microglial conditional knockout of Calhm2 could also rescue MPTP-induced motor defects, DA neuronal loss, and neuroinflammation in PD model mice, suggesting that Calhm2 plays an important role in microglia.

### Neuronal Calhm2 knockout has no effect on TH-positive neuron loss and glial activation

**Figure 3:**
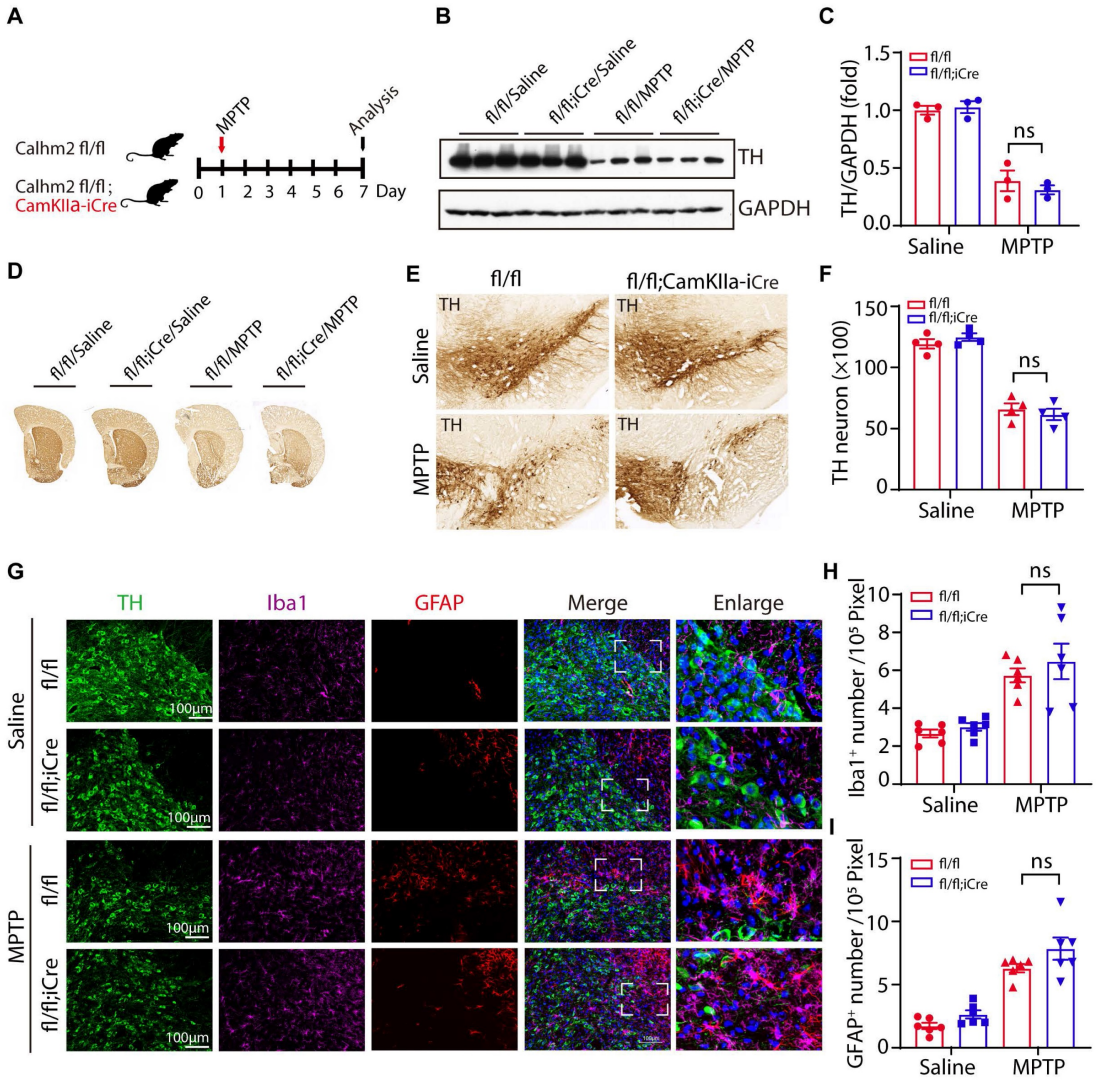
** Neuronal Calhm2 knockout has no effect on TH-positive neuron loss and glial activation.** (A) Experimental flow diagram: Calhm2 flox/flox treated with saline or MPTP vs. Calhm2 flox/flox; CamKIIα-iCre mice (4 intraperitoneal injections of 20 mg/kg at 2 h intervals). Striatal tissue was collected on day 7 after MPTP injection. (B) Immunoblotting of striatal tissue lysates using anti-TH antibody to label DA neurons after MPTP treatment, using anti-GAPDH as a loading control. (C) Statistical analysis of TH expression levels (n = 3). (D) Images of immunohistochemically stained sections of mouse striatum treated with anti-TH antibody. (E) Images of immunohistochemically stained sections of TH in the SNc of mice. (F) Statistical analysis of the number of TH-positive neurons in the SNc of mice represented in A. Data are expressed as mean ± S.E.M. *P < 0.05, **P < 0.01, ***P < 0.001, n = 4. (G) TH (immunostaining) versus Iba1 (immunostaining) and GFAP (immunostaining) positive cells in Calhm2 flox/flox; CamKIIα-iCre mice. (H, I) Statistical analysis of the number of microglia as well as astrocytes. *P < 0.05, **P < 0.01, ***P < 0.001, (n = 6 slices from 3 mice per group).

To test whether neuronal Calhm2 is involved in the MPTP-induced loss of DA neurons and neuroinflammation, the Calhm2 flox/flox mice were crossed with Camkiiα-iCre transgenic mice to obtain a neuronal conditional knockout of Calhm2 mice. We used the same acute MPTP injection model and performed pathological analysis seven days after MPTP injection (**Figure 3A**). We found that the level of TH expression in the striatum was significantly reduced after MPTP treatment, but neuronal conditional Calhm2 knockout failed to rescue its level (**Figure 3B, C**). We next examined neuronal loss using immunohistochemical staining of TH-positive neurons in the striatal and SNc regions and found that neuronal specific knockout of Calhm2 had no protective effect against MPTP-induced neuronal cell loss compared to that of the Calhm2 WT group mice (**Figure 3D-F**).

In addition, we performed immunofluorescence staining and quantification of glial cell numbers in the region of the SNc, and found that neuronal conditional knockout Calhm2 was unable to inhibit either the increase in microglia numbers or astrocyte numbers induced by MPTP treatment (**Figure 3G-I**). Taken together, these results suggest that neuronal Calhm2 knockout has no effect on TH-positive neuron loss and glial activation.

### Calhm2 regulates LPS-induced microglial activation *in vitro*

**Figure 4:**
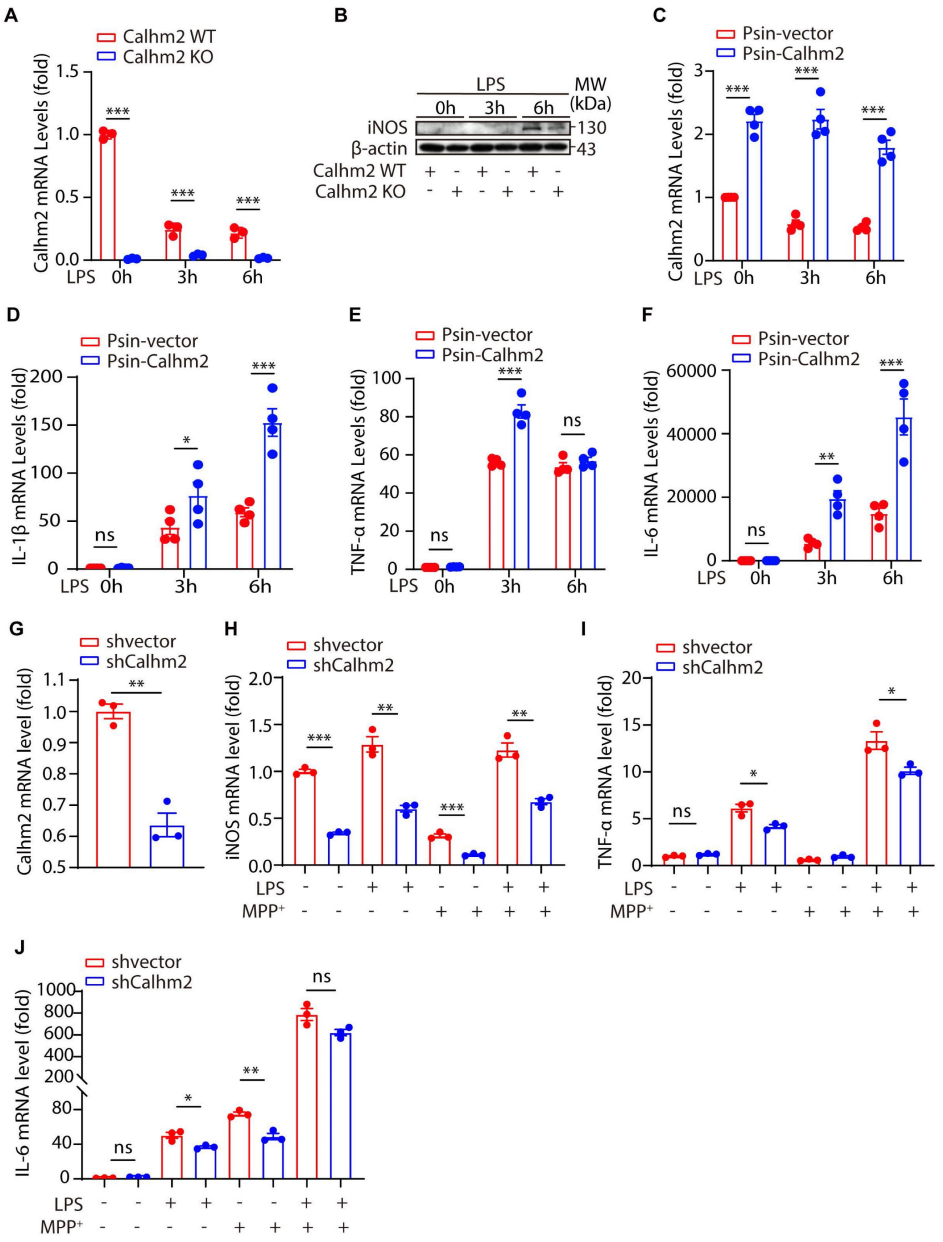
** Calhm2 regulates LPS-induced microglial activation *in vitro*.** (A) Statistical results of Calhm2 expression in Calhm2 WT and Calhm2 knockout mouse primary microglia stimulated with LPS (1 μg/ml) at different time points. (B) Immunoblotting of control and Calhm2 knockout mouse primary microglia after stimulation with LPS (1 μg/ml) at different time points to compare the expression of iNOS in control and Calhm2 knockout group cells. (C-F) Expression of Calhm2, TNFα, IL-1β and IL-6 in control and overexpressed cells by quantitative PCR after LPS stimulation at different time points in IBMDM normal control cells and overexpressed Calhm2 cells. Statistical results are shown as mean ± S.E.M, * *P* < 0.05, ** *P* < 0.01, *** *P* < 0.001. (G-J) Control and Calhm2 knockdown BV2 cells were stimulated with LPS (0.1 μg/ml) and MPP^+^ (1 μg/ml) for 12 h. The expression of Calhm2, TNFα, IL-1β and IL-6 was measured by quantitative PCR and the statistical results showed mean ± S.E.M, *P < 0.05, **P < 0.01, ***P < 0.001.

To further investigate the role of Calhm2 in microglia, we isolated Calhm2 WT and Calhm2 KO primary microglia and stimulated them with lipopolysaccharide (LPS) *in vitro*. Quantitative PCR was used to determine the knockout efficiency of Calhm2, and immunoblotting was used to detect the expression of inducible nitric oxide synthase (iNOS). The results showed that Calhm2 was completely deleted in the Calhm2 KO primary microglia (**Figure 4A**). The expression level of iNOS in WT microglia increased significantly after 6 h of LPS stimulation, whereas the expression level of iNOS in Calhm2 KO microglia was significantly downregulated (**Figure 4B**), indicating that Calhm2 might be involved in the regulation of LPS-induced inflammation. To further investigate the effect of Calhm2 on LPS-induced inflammation levels, we constructed Calhm2 overexpressed plasmid and obtained a stable overexpression of Calhm2 bone marrow-derived macrophage cell line (BMDM). We found that Calhm2 overexpression increased the mRNA levels of TNF-α, IL-1β, and IL-6 induced by LPS stimulation (**Figure 4D-F**), suggesting that overexpression of Calhm2 played a role in promoting LPS-induced inflammation.

To further confirm this, we constructed Calhm2 knockdown plasmid and obtained a stable Calhm2 knockdown BV2 cell line. Consistently, we found that the knockdown of Calhm2 significantly decreased the mRNA levels of TNFα, IL-6, and iNOS under LPS stimulation (**Figure 4G-J**). Interestingly, Calhm2 knockdown also significantly decreased the mRNA levels of iNOS, TNFα, and IL-6 under LPS and MPP^+^ co-stimulation conditions (**Figure 4G-J**), indicating that Calhm2 plays a positive role in the inflammatory response of microglia induced by MPP^+^ and LPS treatment.

### EF-hand domain family member D2 (EFhd2) interacts with Calhm2 in microglia cell line and mouse brain tissue

**Figure 5:**
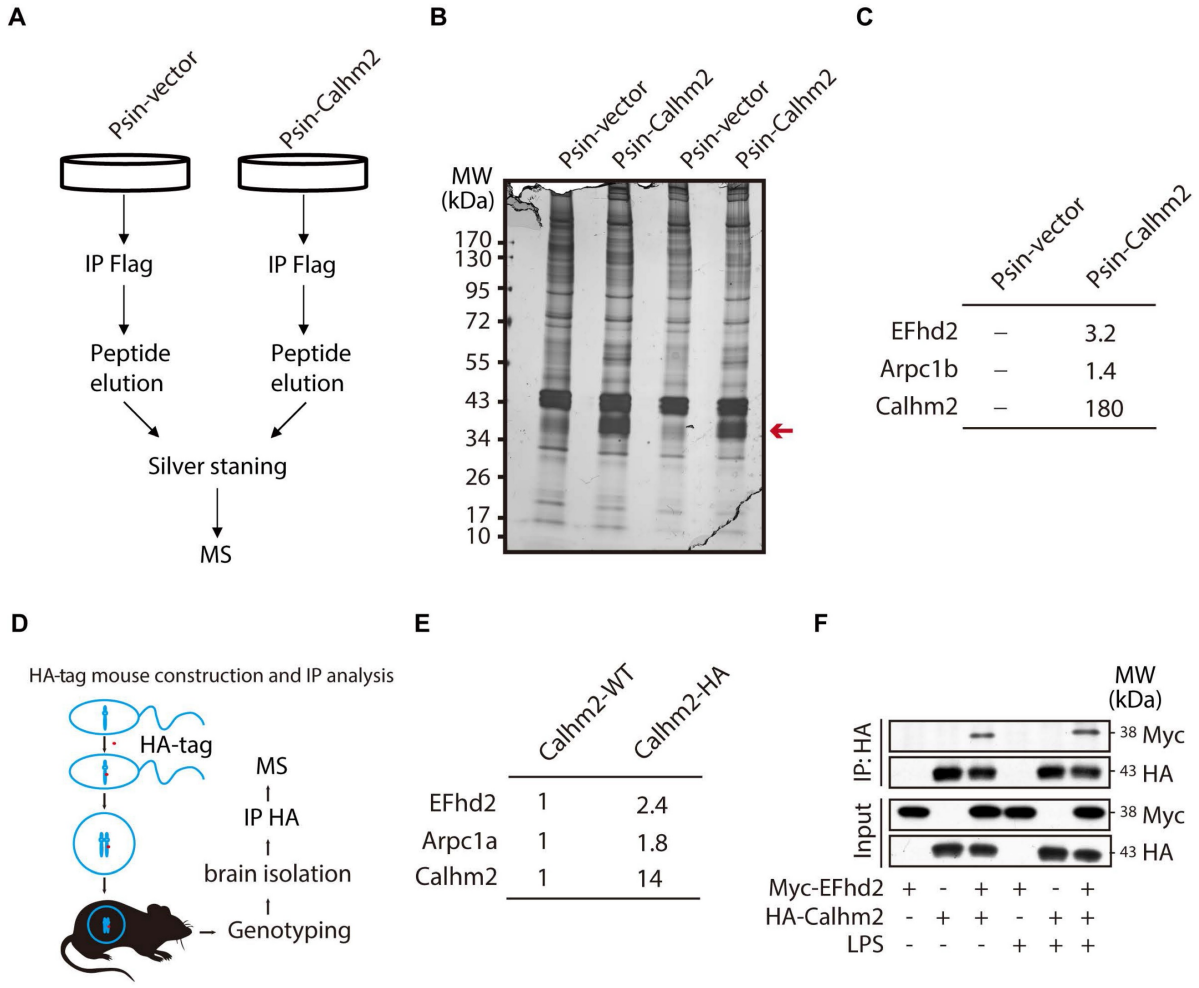
** Calhm2 interacts with EFhd2.** (A) Flow chart of cells collected, immunoprecipitation of Flag, silver stained, and mass spectrometry analysis in BV2 control cells and overexpressed Calhm2 cells; (B) Silver staining results of immunoprecipitation of Flag samples for proteins. (C) Mass spectrometry results analyzing protein peptide segment differences within control and overexpressed Calhm2 cells. (D) Pattern diagram of Calhm2-HA mouse constructs. (E) Immunoprecipitation using Calhm2 WT with Calhm2 HA mouse brain tissues, and mass spectrometry results analyzing Calhm2 HA interacting proteins. (F) Co-transfection of Myc-EFhd2 and HA-Calhm2 plasmids in HEK293T cells, LPS stimulation for 6 h, and immunoblotting for Myc and HA after immunoprecipitation of HA.

Next, to investigate the regulatory mechanism of Calhm2 in microglia, we used BV2 overexpressing Calhm2 cell lines and immunoprecipitated Flag-Calhm2 from the cell lysates and performed a mass spectrometry analysis to identify the possible interacting proteins (**Figure 5A**). The results showed that Calhm2 protein bands were enriched only in the overexpression group (**Figure 5B**). Moreover, the mass spectrometry results showed that EFhd2 and actin-related protein 2/3 complex subunit 1 B (Arpc1B) interacted with Calhm2 (**Figure 5C**). To further identify the interacting protein of Calhm2, we constructed an HA-tag at the C-terminus of the Calhm2 gene and obtained HA-Calhm2 transgenetic mice (**Figure 5D**). Genotyping and HA tag expression are verified in **Figure S3A and S3B**.

To investigate the interaction proteins of Calhm2 in mice brain, the brain tissue of Calhm2 WT and Calhm2-HA mice were lysed and immunoprecipitated, and mass spectrometry analysis was performed to identify possible interacting proteins. Consistently, we found that EFhd2 and Arpc1B also interacted with Calhm2 in mice brain tissue. To further validate the interaction between Calhm2 and EFhd2, we transfected HA-tagged Calhm2 and Myc-tagged EFhd2 plasmids into HEK293T cells and performed immunoprecipitation. We found that EFhd2 interacted with Calhm2 in these cells (**Figure 5F**). Interestingly, a slight increase in the interaction between EFhd2 and Calhm2 occurred under LPS stimulation conditions in the cells.

### Calhm2 regulates the phosphorylation of EFhd2 and the downstream STAT3 signaling in microglia

**Figure 6:**
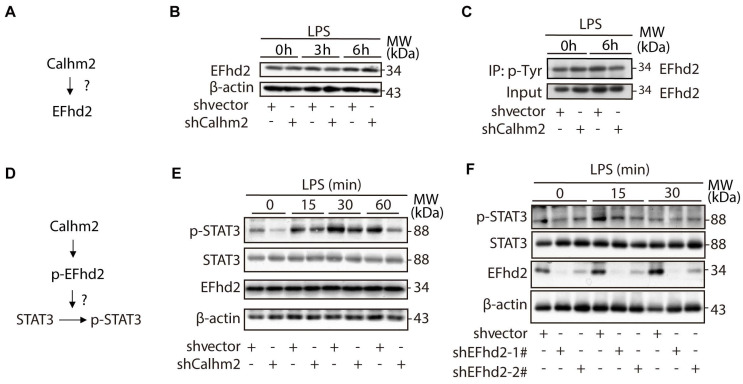
** Calhm2 regulates the phosphorylation of EFhd2 and downstream STAT3 signaling in microglia.** (A) Diagram of the experimental model. (B) Expression of EFhd2 in control and Calhm2 knockdown cells by immunoblotting after LPS stimulation of BV2 normal control cells and knockdown Calhm2 cells at different time points. (C) Immunoprecipitation of phosphotyrosine and immunoblot detection of EFhd2 in BV2 normal control cells and knockdown Calhm2 cells after LPS stimulation for 6 h, (D) Diagram of the experimental model. (E) Expression of EFhd2, p-STAT3 and STAT3 in control and Calhm2 knockdown cells by immunoblotting after LPS stimulation at different time points. (F) Expression of EFhd2, p-STAT3 and STAT3 in control and EFhd2 knockdown cells by immunoblotting after LPS stimulation at different time points.

To investigate the relationship between Calhm2 and EFhd2 (**Figure 6A**), we used an acute LPS-stimulated cellular model in Calhm2 knockdown BV2 cells and found that knockdown of Calhm2 did not change the protein levels of EFhd2 in microglia (**Figure 6B**). Consistently, we found that either Calhm2 knockout or Calhm2 overexpression both failed to impair the EFhd2 protein levels (**Figure S4A and S4B).** It has been reported that tyrosine phosphorylation of EFhd2 is involved in LPS-induced inflammatory response in macrophages 25. Therefore, we performed immunoprecipitation experiments and found that the tyrosine phosphorylated EFhd2 levels were lower in Calhm2 knockdown microglia cells under LPS stimulation conditions, suggesting that Calhm2 might regulate tyrosine phosphorylation of EFhd2 in microglia (**Figure 6C**).

As previously reported, EFhd2 regulates LPS-induced inflammatory response in macrophages by modulating downstream STAT3 signaling 26. To further investigate the role of EFhd2 in LPS-induced inflammatory response and its regulatory mechanism (**Figure 6D**), we examined the downstream phosphorylated STAT3 levels in microglia upon LPS treatment. We found that phosphorylated STAT3 protein levels were largely reduced in Calhm2 knockdown cells upon LPS stimulation (**Figure 6E**). Consistently, we found that intracellular phosphorylated STAT3 levels were significantly reduced in EFhd2 knockdown microglial cells under LPS stimulation (**Figure 6F**). Together, these results suggest that Calhm2 regulates the phosphorylation of EFhd2 and downstream STAT3 signaling in microglia.

### Knockdown of EFhd2 also significantly inhibits LPS-induced inflammation levels in BV2 cells

**Figure 7:**
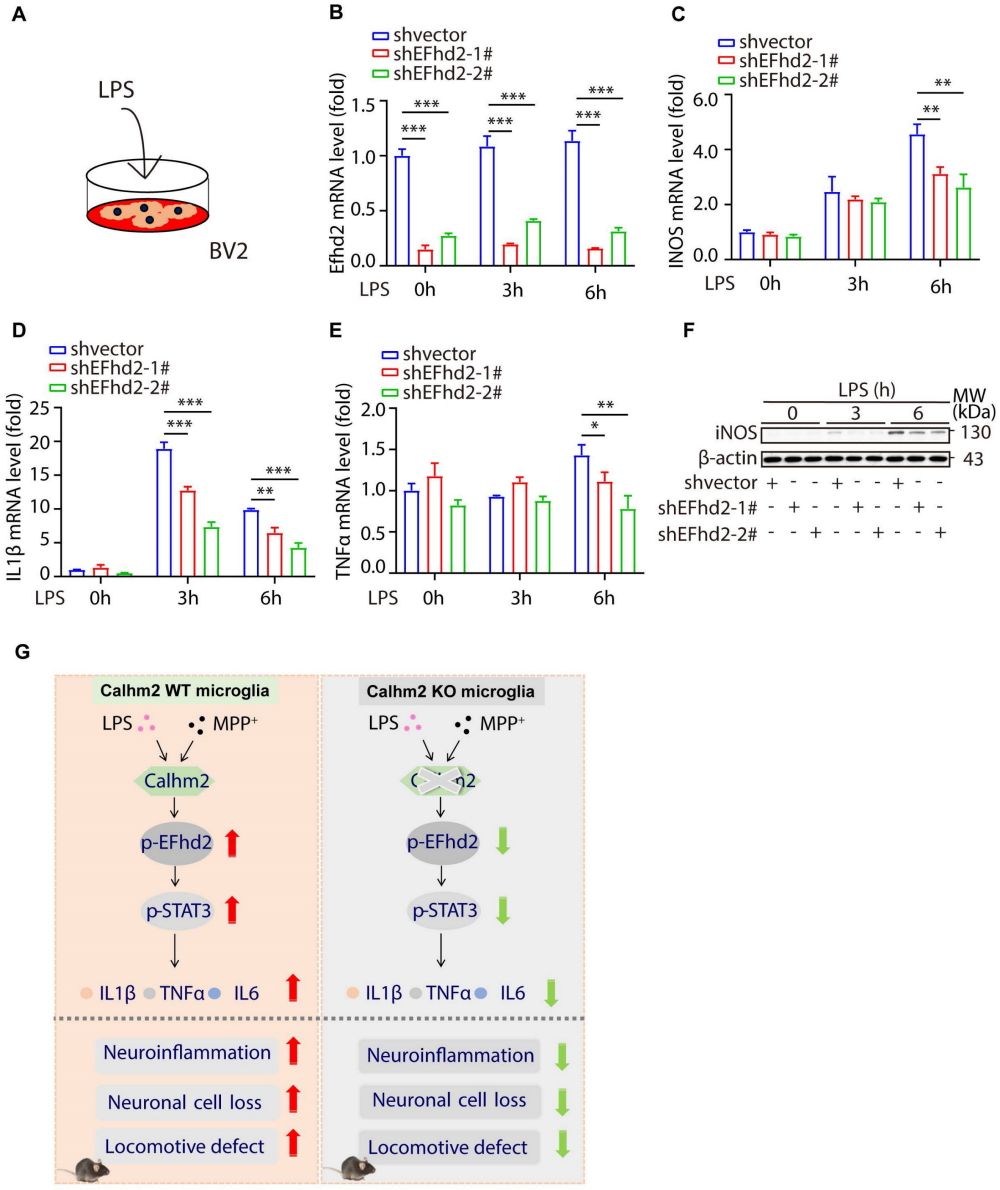
**Knockdown of EFhd2 significantly inhibits LPS-induced inflammation levels in BV2 cells.** (A) Diagram of the experimental model. (B-E) Quantitative PCR of expression of EFhd2, iNOS, TNFα and IL-1β in control and knockdown EFhd2 cells after LPS stimulation at different time points; statistical results are shown as mean ± S.E.M, *P < 0.05, **P < 0.01, ***P < 0.001. (F) Expression of iNOS and EFhd2 in control and knockdown EFhd2 cells was measured by immunoblotting after LPS stimulation at different time points, using anti-β-actin as a loading control. (G) The working model of microglial Calhm2 in PD mouse model.

To investigate the role of EFhd2 in LPS-induced cellular inflammation, we used stable knockdown EFhd2 microglial cell lines and treated them with LPS (**Figure 7A**). We found that the knockdown efficiency of EFhd2 was high in the two stable knockdown cell lines (**Figure 7B**). Moreover, intracellular inflammatory factors such as iNOS, TNFα, and IL-1β were significantly increased upon LPS stimulation, whereas knockdown of EFhd2 significantly suppressed these inflammatory cytokine levels (**Figure 7C-F**). Together, these results suggest that EFhd2 also plays a positive role in LPS-induced microglial inflammation levels.

In summary, we demonstrated that Calhm2 plays an important role in the MPTP-induced PD mouse model. Both conventional and microglial conditional knockout of Calhm2 rescued MPTP-induced TH-positive neuronal loss and inhibited neuroinflammation, thereby improving motor deficits in PD model mice. Mechanistically, we found that Calhm2 interacted with EFhd2 and regulated downstream STAT3 signaling in microglia, presenting a potential therapeutic target for microglia-mediated neuroinflammation-related diseases (**Figure 7G**).

## Discussion

Over the past decades, age, genetics, environment, immune status, and sex have been widely discussed as important factors in the development of PD. However, the molecular mechanisms underlying PD pathogenesis are not yet fully understood. Neuroinflammation caused by neuroglia (astrocytes and microglia) present in the brain is a common feature of PD 27. In our previous study, we found that defective microglial autophagy accelerates inflammatory vesicle activation in mice, causing PD-like symptoms 28. Therefore, targeting microglia-mediated neuroinflammation for the treatment of central nervous system disorders is an emerging remedy. In this study, we used conventional Calhm2, neuronal Calhm2, and microglial Calhm2 knockout mice to explore the role of Calhm2 in Parkinson's disease. We found that both conventional and microglial conditional Calhm2 knockout significantly reduced DA neuronal loss and microglial numbers, thereby improving locomotor performance in PD model mice. Thus, our results strongly support the important role of Calhm2 in PD, possibly through the modulation of microglial activation.

Calcium homeostasis in cells was an important factor in the development of neuroinflammation, and dysregulation of Ca^2+^ homeostasis was also frequently observed in models of sporadic and familial PD 29. More importantly, clinical trials showed that dihydropyridine calcium channel blocker (DiCCB) exposure was associated with a reduced risk of PD incidence, particularly in older patients, and with reduced mortality among patients with PD 30. Inhibition of TRPM2 by AG490 has been reported to be neuroprotective in a PD Animal Model 31. Meanwhile, calcium-sensing receptors have been reported to activate the NLRP3 inflammasome by enhancing intracellular calcium levels and inhibiting cAMP levels 32. Moreover, blocking calcium channels with Nicardipine significantly inhibited microglial activation *in vitro* 33. In this study, we found that both knockout of Calhm2 and knockdown of Calhm2 significantly reduced LPS-induced inflammatory cytokine levels, whereas overexpression of Calhm2 significantly increased LPS-induced inflammation, suggesting that Calhm2 plays an important role in microglia-mediated neuroinflammation. This finding is consistent with previous studies showing that reducing intracellular calcium ion concentrations by inhibiting calcium channel proteins contributes to lower levels of neuroinflammation 24. Our results suggest that targeting Calhm2 could be a possible way to reduce neuroinflammation and thereby improve neurological disease.

Calhm2, a membrane protein, has been shown to mediate the transport of calcium and adenosine triphosphate (ATP), regulating both intra- and extracellular calcium and ATP concentrations 34-36. Our previous study showed that Calhm2 is abundantly expressed in the central nervous system, and knockout of microglial Calhm2 in AD model mice improved cognitive memory and pathological performance 24. However, the mechanism through which Calhm2 regulates microglial activation remains unclear. In this study, we found that Calhm2 interacted with EFhd2, a calcium-binding protein that possesses two EF chiral structures that bind calcium ion to maintain the normal conformation and the function 37. A previous study showed that knockdown of EFhd2 significantly reduced LPS-induced inflammation in macrophages; however, the role of EFhd2 in microglia remains unclear 25. In this study, we found that EFhd2 knockdown in microglia also significantly suppressed LPS-induced inflammation, which was achieved by regulating downstream STAT3 signaling. Overactivation of STAT3 has been reported to lead to the secretion of pro-inflammatory factors in microglia and promote neuronal loss 38, suggesting that tight regulation of STAT3 function is critical to the pathology of PD. We also found that knockdown of either Calhm2 or EFhd2 significantly reduced LPS-induced STAT3 activation, suggesting that Calhm2 may regulate STAT3 signaling through interaction with EFhd2 in microglia.

In conclusion, we demonstrated that microglial Calhm2 is involved in microglia-mediated neuroinflammation and the pathogenesis of PD by modulating the EFhd2-STAT3 pathway in microglia, which provides potential therapeutic targets for microglia-mediated neuroinflammation-related diseases.

## Materials and methods

### Mice

The Calhm2 mice used in this experiment have been described in our previous study 24. Mice were housed in an SPF-rated animal house, provided with a constant temperature of 20-23 °C, maintained on a 12-h diurnal cycle, and provided with an ad libitum diet and water. All animal experiments were approved by the Institutional Animal Care and Use Committee of Beijing Institute of Basic Medical Sciences.

Tamoxifen (Sigma-Aldrich, catalogue no. T5648) was dissolved in corn oil (Sigma-Aldrich, catalogue no. C8267). Intragastric tamoxifen (20 mg) was administered to the mice on three consecutive days.

### Calhm2-HA transgenetic mice

An HA-tag was added to the C-terminus of the mouse Calhm2 gene. Calhm2-C-WT and Calhm2-C-HA PCR primers were used to distinguish wild-type, heterozygous, and homozygous.

Calhm2-WT: F: GCTCACTGCTTAAGAGCCTG; R: TCCCAGTGGGTTGTCAACAG; Calhm2-HA: F: GTGTTCCTGACCAAGTGCCT; R: ATCCGGCACATCATACGGAT (95 ℃ 7 min; 95 ℃ 15 s, 60 ℃ 20 s, 72 ℃ 30 s, 35 Cycle; 72 ℃ 10 min; 4 ℃).

### Rotarod test

An accelerated rotating rod (Panlab, LE8200; Energia, Cornella, Spain) was used to evaluate motor impairment in mice. For training, mice were placed on the rod at a constant speed of 10 rpm, 3 times a day (1 h interval) for 3 days prior to the test. During the test, the rod was accelerated from 4 rpm to 40 rpm for 300 s. The time spent on the rod was recorded for each mouse, each mouse was tested three times at 1 h intervals and the average of the three tests was taken as the mean latency for each mouse.

### Cell culture and stable cell line

DMEM basic medium and penicillin-Streptomycin were purchased from Life technologies corporation and Fetal Bovine Serum (FBS) was purchased from Biological Industries. Cells were passaged when the growth density of adherent cells reached 70-90%. We add 1 ml of 0.05% trypsin, in a semi-suspension state and used 1 ml of culture solution (10% FBS and, 1% penicillin-Streptomycin) to terminate the digestion, we then centrifuged at 800 rmp for 5 min and aspirated off the supernatant. 1/3 of the cells was incubated at 37 °C in an incubator with 5% CO_2_ cells.

### Isolation of microglia

Microglia were isolated and purified as described in previous studies 24. Briefly, the mice were anesthetized by intraperitoneal injection of sodium pentobarbital (70 mg/kg, dissolved in saline). The whole brain tissue was isolated, scalpel cut into small pieces, suspended in Dounce buffer [1.5 mM Hepes and 0.5% glucose in hanks' balanced salt solution (HBSS) buffer] and gently homogenized using a homogenizer. After blowing the cells with phosphate-buffered saline buffered salt solution [PBS; NaCl (8 g/l), KCl (0.2 g/l), Na2HPO4 (1.44 g/l), and KH2PO4 (0.24 g/l)], they were passed through a 70 μm filter; myelin proteins were then removed by differential centrifugation with cell separator Percoll to obtain a single cell suspension; erythrocyte lysate was used to remove hemoglobin; microglia were purified using Miltenyi's CD11b magnetic beads (Miltenyi Biotec, 130-093-634).

### Immunohistochemistry and Immunofluorescence

All procedures were performed as described in previous studies 28. Briefly, mice were perfused with saline and their brains were fixed with 4% paraformaldehyde (w/v). The fixed mouse brains were cryoprotected in 30% sucrose. Coronal sections were cut to a thickness of 40 μm and stained with GFAP (1:1000; Sigma-Aldrich, catalogue no. G3893), Iba1 (1:500; NOVUS; NB100-1028), TH (1:500; Pel-Freez, catalogue no. P40101-150). Coronal sections were incubated with rabbit polyclonal anti-TH (1:500, Pel Freez Biologicals, P40101, Rogers, AR, USA) followed by a horseradish peroxidase (HRP) reaction using the ABC method (VECTASTAIN ABC Kit, Rabbit, IgG, ZF0328). Signal amplification was carried out at a ratio of 1:100 between A and B liquids. The reaction was carried out at room temperature for 30 min, visualized using a DAB kit (1:20, ORIGENE, ZLI-9019), and scanned using a digital pathology section scanning analysis system. Immunohistochemistry (IHC) quantification was performed using Image-Pro Plus software (Media Cybernetics Inc.). To quantify the Iba1 or GFAP IHC levels, we calculated all intact cells in the same pixel region in the indicated brain regions.

### Immunoprecipitation and Immunoblot Analysis

Immunoprecipitation and immunoblot analyses were performed as described in previous study 24. Briefly, tissues for co-immunoprecipitation were lysed in a buffer containing 50 mM Nacl (pH 7.4), 1 mM EDTA, 1 mM EGTA, and 0.05% Triton-100. Prior to immunoprecipitation, the tissue was lysed to remove the supernatant and pre-incubated with protein G agarose beads for 1 h at 4 °C. After removal of the beads by centrifugation, the lysates were incubated with the appropriate antibody in the presence of 25 μl protein G agarose beads for at least 3 h at 4 °C. After three washes, protein expression was detected by western blotting of cell lysates or immunoprecipitates using the appropriate antibodies; that is, the proteins were separated on a 10% polyacrylamide gel and transferred to NC membranes. The membranes were incubated in Tris-buffered saline containing 5% milk and Tween-20 for 1 h, followed by incubation with primary antibody overnight at 4 °C. After incubation with goat anti-mouse or goat anti-rabbit HRP-coupled secondary antibodies (GE Healthcare) for 1 h, the indicated protein was detected using an enhanced chemiluminescence western blotting system (Amersham Biosciences).

### Quantitative RT-PCR

Total RNA was extracted from the brain tissue or cells using TRIzol reagent (Invitrogen, catalogue no. 15596018). One microgram of RNA was used as a template for the one-step first-strand cDNA synthesis kit (AT341). PCR amplification was performed using a 2 × SYBR Green PCR master mix (TransGen Biotech, catalogue number: AQ131) and an Agilent Mx3005P RT-PCR system, and gene expression was detected. The primers used for the analysis are listed in **Supplementary Table 1**. The mRNA levels of the tested genes were normalized to that of β-actin.

### Statistical Analysis

Image Pro and GraphPad software were used for data statistics and analysis. Unpaired Students and two-tailed t test, and two-way ANOVA were used to determine significance between the data. In all experiments, *P* < 0.05 was considered a statistically significant difference.

## Supplementary Material

Supplementary figures and table.

## Abbreviations

AD: Alzheimer's disease
ATP: adenosine triphosphate
Arpc1B: actin-related protein 2/3 complex subunit 1 B
BMDM: bone marrow-derived macrophage
CALHM: calcium homeostasis modulator
Calhm2 KO: Calhm2 knockout
Calhm2 WT: Calhm2 wide-type
DA: dopaminergic
EFhd2: EF-hand domain family member D2
FPS: Fetal Bovine Serum
GFAP: glial fibrillary acidic protein
HRP: horseradish peroxidase
Iba1: ionized calcium-binding adaptor molecule 1
IHC: immunohistochemistry
iNOS: inducible nitric oxide synthase
LPS: lipopolysaccharide
MPTP: 1-Methyl-4-phenyl-1,2,3,6-tetrahydropyridine
PD: Parkinson's disease
SNc: substantia nigra
TH: tyrosine hydroxylase
