# Supplementary material for: Deletion of Calhm2 alleviates MPTP-induced Parkinson's disease pathology by inhibiting EFHD2-STAT3 signaling in microglia
Source: Theranostics. 2023 Mar 13;13(6):1809–22. doi: 10.7150/thno.83082 (PMC10091876; doi:10.7150/thno.83082)
Supplement: Supplementary file 1 — Supplementary figures and table. [file thnov13p1809s1.pdf]

Supplementary Figure legends:

Figure S1: The expression of Calhm2 in Calhm2 WT and Calhm2 KO mice brain tissue. (A) Quantitative PCR of expression of Calhm2 in Calhm2 WT and Calhm2 KO mice brain tissue. Statistical results are shown as mean ± S.E.M, $*P < 0.05$, $**P < 0.01$, $***P < 0.001$.

Figure S2: The expression of Calhm2 in isolated microglia from Calhm2 flox/flox mice and Calhm2 flox/flox;Cx3cr1-CreER mice. (A) Quantitative PCR of expression of Calhm2 in isolated microglia from Calhm2 flox/flox mice and Calhm2 flox/flox;Cx3cr1-CreER mice. Statistical results are shown as mean ± S.E.M, $*P < 0.05$, $**P < 0.01$, $***P < 0.001$.

Figure S3: The genotyping and immunoprecipitation of HA-Calhm2 in mouse brain tissue. (A) The genotyping result of Calhm2 WT mice and Calhm2-HA mice. (B) The brain tissue from Calhm2 WT mice and Calhm2-HA mice were lysed and immunoprecipited with HA antibody, and then immunoblotting with anti-HA antibody.

Figure S4: Calhm2 does not impair EFhd2 protein levels. (A) Expression of EFhd2 in Calhm2 WT and Calhm2 KO primary microglia by immunoblotting after LPS stimulation at different time points. (B) Expression of EFhd2 in control and Calhm2 overexpression IBMDM cells after LPS stimulation at different time points.

A

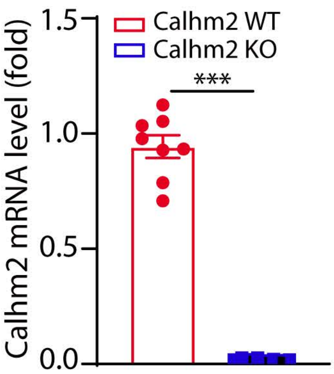

A

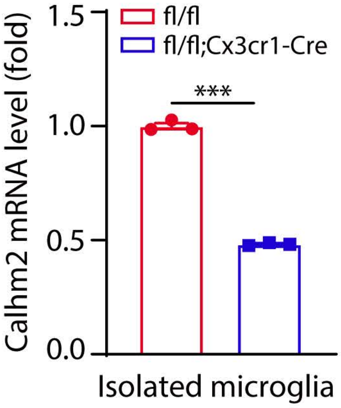
fl/fl
fl/fl;Cx3cr1-Cre
Calhm2 mRNA level (fold)
1.5
1.0
0.5
0.0
***
Isolated microglia

A

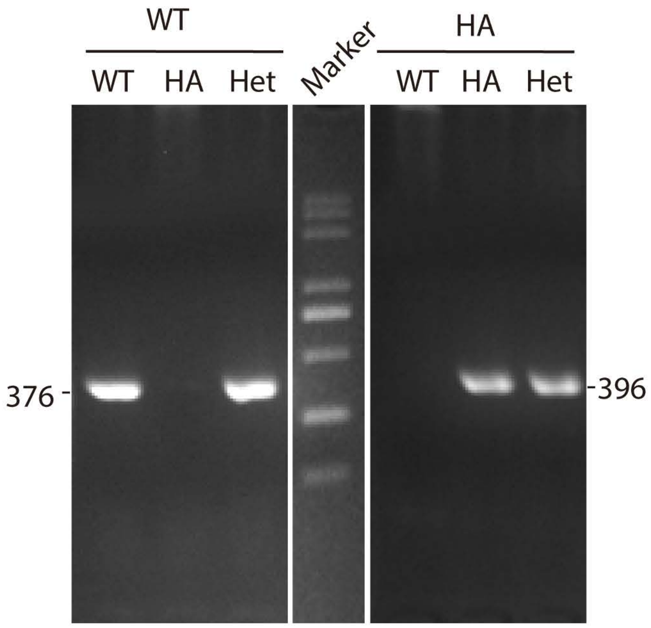
WT
WT
HA
Het
Marker
HA
WT
HA
Het
376
396

B

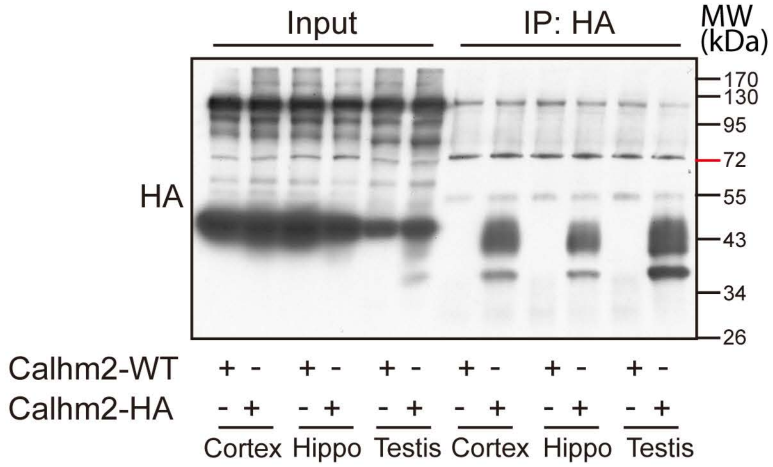
Input
IP: HA
MW (kDa)
170
130
95
72
55
43
34
26
HA
Calhm2-WT
Calhm2-HA
Cortex
Hippo
Testis
Cortex
Hippo
Testis

A

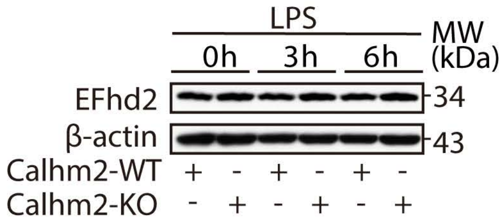
LPS
0h
3h
6h
MW
(kDa)
EFhd2
β-actin
34
43
Calhm2-WT + - + - + -
Calhm2-KO - + - + - +

B

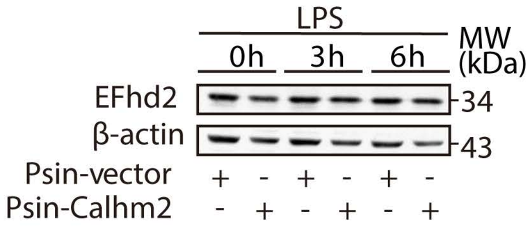
LPS
0h
3h
6h
MW
(kDa)
EFhd2
β-actin
34
43
Psin-vector + - + - + -
Psin-Calhm2 - + - + - +

Supplementary Table 1

Reagents or resource	Source	Identifier
**Antibody**		
TH	Pel-Freez	P40101-150
TH	Cell Signaling Technology	2792
Iba1	NOVUS	NB100-1028
Iba1	WAKO	NCNP24
GFAP	Sigma	G3893
EFhd2	ZEN-BIOSCIENCE	163761
Stat3	ZEN-BIOSCIENCE	251219
p-stat3	ZEN-BIOSCIENCE	381552
HA	Cell Signaling Technology	3724S
Myc	abcam	Ab32
Photyrosine	MILLIPORE	16-454
β-actin	Proteintech	66009-1-lg
GAPDH	Cwbiotech	CW0100A
**Chemicals & probes**		
Tamoxifen	Sigma-Aldrich	T5648
Corn oil	Sigma-Aldrich	C8267
MPTP	Sigma-Aldrich	M0896-100MG
LPS	Merck Millipore	LPS25
MPP^+	Sigma-Aldrich	D048-100MG
Triton X-100	Sigma-Aldrich	9036-19-5
CD11b MicroBeads	Miltenyi Biotec	130-093-634
HA beads	Bimake	B26201
Protein A/G	Bimake	B23201
Myc beads	Bimake	B26301
**Primers**		
Mouse *Calhm2*	Forward	5’-CCTGACCAAGTGCCTCAAACA-3’

	Reverse	5’-GGTCCTCGTTGGTACGGTACTG-3’
Mouse *Tnf-α*	Forward	5’-CAGGCGGTGCCTATGTCTC-3’
	Reverse	5’-CGATCACCCCGAAGTTCAGTAG-3’
Mouse *Inos*	Forward	5’-GTTCTCAGCCCAACAATACAAGA-3’
	Reverse	5’-GTGGACGGGTCGATGTCAC-3’
Mouse *Il-1β*	Forward	5’-TGTAATGAAAGACGGCACACC-3’
	Reverse	5’-TCTTCTTTGGGTATTGCTTGG-3’
Mouse *Il-6*	Forward	5’-GCTACCAAACTGGATATAATCAGGA-3’
	Reverse	5’-CCAGGTAGCTATGGTACTCCAGAA-3’
Mouse *β-actin*	Forward	5’-GGCTGTATTCCCCTCCATCG-3’
	Reverse	5’-CCAGTTGGTAACAATGCCATGT-3’
shCalhm2	Forward	5’-CCGGGTCTGTGCTCTCAGTGAGTTTCTCGAGAAACTCACTGAGAGCACAGACTTTTTTG-3’
	Reverse	5’-AATTCAAAAAGTCTGTGCTCTCAGTGAGTTTCTCGAGAAACTCACTGAGAGCACAGAC-3’
shEFhd2-1	Forward	5’-CCGGCGTTTGCCTCAGCGGATACTCGAGTATCCGCTGAGGCA AACGTTTTTG-3’
	Reverse	5’-AATTCAAAAACGTTTGCCTCAGCGGATACTCGAGTATCCGCTG AGGCAAACG-3’
shEFhd2-2	Forward	5’-CCGGGAGAAGATGTTCAAGCAGTATCTCGAGATACTGCTTGAACATCTTCTCTTTTTG-3’
	Reverse	5’-AATTCAAAAAGAGAAGATGTTCAAGCAGTATCTCGAGATACTGCTTGAACATCTTCTC-3’
